# Complete Mitochondrial Genome Sequencing Reveals Novel Haplotypes in a Polynesian Population

**DOI:** 10.1371/journal.pone.0035026

**Published:** 2012-04-13

**Authors:** Miles Benton, Donia Macartney-Coxson, David Eccles, Lyn Griffiths, Geoff Chambers, Rod Lea

**Affiliations:** 1 School of Biological Science, Victoria University of Wellington, New Zealand; 2 Kenepuru Science Centre, Institute of Environmental Science and Research, Sandringham, New Zealand; 3 Genomics Research Centre, Griffith Health Institute, Griffith University, Queensland, Australia; Erasmus University Medical Center, The Netherlands

## Abstract

The high risk of metabolic disease traits in Polynesians may be partly explained by elevated prevalence of genetic variants involved in energy metabolism. The genetics of Polynesian populations has been shaped by island hoping migration events which have possibly favoured thrifty genes. The aim of this study was to sequence the mitochondrial genome in a group of Maoris in an effort to characterise genome variation in this Polynesian population for use in future disease association studies. We sequenced the complete mitochondrial genomes of 20 non-admixed Maori subjects using Affymetrix technology. DNA diversity analyses showed the Maori group exhibited reduced mitochondrial genome diversity compared to other worldwide populations, which is consistent with historical bottleneck and founder effects. Global phylogenetic analysis positioned these Maori subjects specifically within mitochondrial haplogroup - B4a1a1. Interestingly, we identified several novel variants that collectively form new and unique Maori motifs – B4a1a1c, B4a1a1a3 and B4a1a1a5. Compared to ancestral populations we observed an increased frequency of non-synonymous coding variants of several mitochondrial genes in the Maori group, which may be a result of positive selection and/or genetic drift effects. In conclusion, this study reports the first complete mitochondrial genome sequence data for a Maori population. Overall, these new data reveal novel mitochondrial genome signatures in this Polynesian population and enhance the phylogenetic picture of maternal ancestry in Oceania. The increased frequency of several mitochondrial coding variants makes them good candidates for future studies aimed at assessment of metabolic disease risk in Polynesian populations.

## Introduction

**Figure 1:**
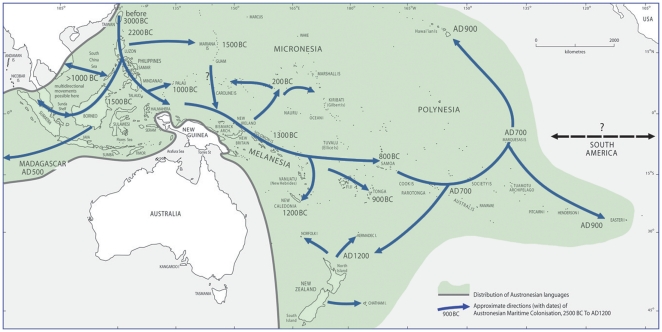
Map outlining migratory paths of Austronesian speaking populations, including estimated dates. Adapted from Bellwood *et al*., (2011) [52].

Scientific evidence from linguistics, archaeology and genetics indicates that the Maori population of New Zealand (NZ) represents the final link in a long chain of island-hopping voyages by Polynesians, which began in Taiwan and stretched through Melanesia and across the Pacific Islands over a period of 5–6000 years (Figure 1). Around 800 years ago one or more small groups of voyagers arrived in NZ from Tahiti, via the Cook Islands. This event marked the last of the great human migrations and the creation of an isolated founder population. The widespread intermarriage between Maoris and Europeans over the past 200 years (8–10 generations) has introduced substantial European genomic ancestry (∼40%) into the contemporary Maori gene pool [1].

Maoris, and Polynesians more generally, are disproportionately affected with certain metabolic disease traits eg. obesity and type 2 diabetes mellitus [2], [3], [4], [5]. Given that these traits are partially influenced by genetic factors it is likely that genes involved in energy metabolism play a role in disease risk [6]. Mitochondrial genes could potentially account for some of the high prevalence of metabolic disease traits in Maoris. Coding variants in mitochondrial genes that exhibit unusually high frequencies in Maori may have been driven to high frequency by positive selection due to periods of feast and famine during the migrations (ie. thrifty genes) [7]. Alternatively, these mitochondrial variants may have simply increased in frequency in Maoris via genetic drift as a consequence of repeated founder events and subsequent population bottlenecks.

Complete mitochondrial genome sequence data have been previously investigated to elucidate the evolutionary history among human populations around the world [8]. Studies have also comprehensively investigated mitochondrial genome variation in relation to metabolic syndrome traits [9], [10]. However, no complete mitochondrial genome surveys have involved a Maori sample and Polynesians more generally have been under-represented. Given the unusual maternal history of Maoris it is likely that a unique mitochondrial genomic makeup exists in this Polynesian subgroup. In this study we sequenced the entire mitochondrial genome in a group of Maori individuals and performed population genetic analyses to characterise the patterns of genomic variation in this Polynesian population. These new data provide the opportunity to enhance the phylogenetic picture of the mitochondrial genome in the South Pacific region and establish a foundation for future studies of mitochondrial DNA and metabolic disease traits in Polynesian populations.

## Results

### Complete mtDNA sequences and genetic diversity

A summary of mitochondrial (mt) sequence variation for all 20 Maori mtDNA genomes is shown in Table 1. These sequences are the only complete NZ Maori sequences currently available (at time of writing). Previous studies have suggested that there is very limited mtDNA variation in Polynesians in general, and even less in Maori [11], [12] and no Maori-specific genetic mtDNA markers have yet been identified.

**Table 1:** Variation across 20 complete Maori mtDNA sequences.

	HVRII				12SrRNA			16SrRNA				ND1	ND2		COI					ATP6			
Position	73	146	151	263	750	1185	1438	1692	1806	2416	2706	3909	4769!	5465	6261	6719	6782	6905	7028	8860	8865	9123	9145
CRS*	A	T	C	A	A	C	A	A	T	T	A	C	A	T	G	T	T	A	C	A	G	G	G
mt3	G	C	.	G	G	T	G	.	.	.	G	.	.	C	.	C	.	.	T	G	.	A	.
mt6	G	C	.	G	G	.	G	.	.	.	G	.	G	C	.	C	.	.	T	G	.	A	.
mt8	G	C	.	G	G	T	G	.	.	.	G	.	.	C	.	C	.	.	T	G	.	A	.
mt9	G	C	.	G	G	.	G	.	.	.	G	T	G	C	.	C	.	.	T	G	.	A	.
mt10	G	C	T	G	G	.	G	G	.	C	G	.	G	C	.	C	.	.	T	G	.	A	.
mt11	G	C	.	G	G	.	G	.	.	.	G	.	G	C	.	C	.	.	T	G	A	A	.
mt12	G	C	.	G	G	.	G	.	.	.	G	.	G	C	.	C	.	.	T	G	A	A	.
mt13	G	C	.	G	G	T	G	.	.	.	G	.	.	C	.	C	.	.	T	G	.	A	.
mt14	G	C	.	G	G	T	G	.	.	.	G	.	.	C	.	C	.	.	T	G	.	A	.
mt16	G	C	.	G	G	.	G	.	.	.	G	.	G	C	.	C	.	.	T	G	.	A	.
mt18	G	C	.	G	G	.	G	.	.	.	G	.	G	C	A	C	.	G	T	G	.	A	A
mt19	G	C	.	G	G	.	G	.	.	.	G	T	G	C	A	C	.	.	T	G	.	A	.
mt21	G	C	.	G	G	.	G	.	.	.	G	T	G	C	.	C	.	.	T	G	.	A	.
mt23	G	C	.	G	G	.	G	.	.	.	G	.	G	C	.	C	.	.	T	G	.	A	.
mt24	G	C	.	G	G	.	G	.	.	.	G	.	G	C	.	C	.	.	T	G	.	A	.
mt25	G	C	.	G	G	T	G	.	.	.	G	.	.	C	.	C	.	.	T	G	.	A	.
mt26	G	C	.	G	G	.	G	.	.	.	G	.	G	C	.	C	C	.	T	G	.	A	.
mt28	G	C	.	G	G	T	G	.	.	.	G	.	.	C	.	C	.	.	T	G	.	A	.
mt29	G	C	.	G	G	T	G	.	C	C	G	.	.	C	.	C	.	.	T	G	.	A	.
mt30	G	C	T	G	G	.	G	G	.	.	G	.	G	C	.	C	.	.	T	G	.	A	.
n (variant)	*20*	*20*	*2*	*20*	*20*	*7*	*20*	*2*	*1*	*2*	*20*	*3*	*13*	*20*	*2*	*20*	*1*	*1*	*20*	*20*	*2*	*20*	*1*
Amino-acid change												syn	syn	syn	A120T	syn	syn	syn	syn	T112A	syn	syn	A207T
Conservation Index												8	28	51	97	100	100	97	100	72	92	100	100
Protein Position												201	100	332	120	272	293	334	375	112	113	199	207

COIII		ND3	ND4	S(AGY)	ND5	Cyt b				NC		D-loop & HVRI								
9255	9722	10238	11719	12239	14022	14766	15014	15326	15746	16051	16086	16126	16189	16217	16243	16247	16260	16261	16295	16519
C	T	T	G	C	A	C	T	A	A	A	T	T	T	T	T	A	C	C	C	T
.	.	C	A	T	G	T	.	G	G	.	.	.	C	C	.	G	.	T	.	C
.	.	C	A	T	G	T	.	G	G	.	.	C	C	C	.	.	.	T	.	C
.	.	C	A	T	G	T	.	G	G	.	.	.	C	C	.	G	.	T	.	C
.	.	C	A	T	G	T	.	G	G	.	.	C	C	C	.	G	.	T	.	C
.	.	C	A	T	G	T	.	G	G	.	.	C	C	C	.	.	.	T	.	C
.	.	C	A	T	G	T	.	G	G	.	.	C	C	C	.	.	.	T	.	C
.	.	C	A	T	G	T	.	G	G	.	.	C	C	C	.	.	.	T	.	C
.	.	C	A	T	G	T	.	G	G	.	.	.	C	C	.	G	.	T	+A	C
.	.	C	A	T	G	T	.	G	G	.	.	.	C	C	.	G	.	T	.	C
.	.	C	A	T	G	T	C	G	G	.	.	.	C	C	.	G	T	T	.	C
.	.	C	A	T	G	T	.	G	G	G	C	.	C	C	.	G	.	T	.	C
.	.	C	A	T	G	T	.	G	G	.	.	C	C	C	.	G	.	T	.	C
.	.	C	A	T	G	T	.	G	G	.	.	C	C	C	.	.	.	T	.	C
.	.	C	A	T	G	T	.	G	G	.	.	C	C	C	.	.	.	T	.	C
.	.	C	A	T	G	T	.	G	G	.	.	C	C	C	.	G	.	T	.	C
.	.	C	A	T	G	T	.	G	G	.	.	.	C	C		G	.	T	.	C
T	.	C	A	T	G	T	.	G	G	.	.	.	C	C	C	G	.	T	.	C
.	.	C	A	T	G	T	.	G	G	.	.	.	C	C	.	G	.	T	.	C
.	C	C	A	T	G	T	.	G	G	.	.	.	C	C	.	G	.	T	.	C
.	.	C	A	T	G	T	.	G	G	.	.	C	C	C	.	G	.	T	.	C
*1*	*1*	*20*	*20*	*20*	*20*	*20*	*1*	*20*	*20*	*1*	*1*	*10*	*20*	*20*	*1*	*14*	*1*	*20*	*1*	*20*
P17S	syn	syn	syn		syn	T7I	F90L	T194A	I334V											
100	100	100	97		44	49	90	21	26											
17	172	60	320		562	7	90	194	334											

* CRS = Cambridge Reference Sequence, Boldface positions represent rare variants in the CRS. As per phylotree nomenclature, variants toward a base identical-to-state to the CRS are indicated with !

Sequence variation was identified by comparison against the revised Cambridge Reference Sequence (CRS) [13], which belongs to haplogroup H (commonly found in European peoples). The mt sequence variation identified in the Maori individuals differed from the CRS at 44 variable sites (see Table 1). Of these variant sites, 22 were fixed in all 20 Maori individuals – these are the defining markers of mitochondrial haplogroup B, and its further substructure (haplotypes) such as B4a1a1, to which Maoris belong. There were 12 singleton variants identified and a further 10 variants were shared by two or more individuals and define subclades within the Maori mtDNA phylogeny.

The limited sequence variation was validated by calculation of h and π diversity statistics in DNAspV5 [14]. Table 2 shows the amount of DNA sequence diversity of 189 complete mtDNA sequences as well as diversity within each specific population. The Maori group were found to exhibit high haplotype diversity (h = 0.92), yet diversity was substantially lower than that seen in any of the other three populations (see Table 2). When looking at the nucleotide (π) diversity it can be seen that Maoris exhibit a value 10-fold lower (π = 0.00018) compared to that of the other populations. As expected there is no maternal European admixture identified in this group. All mtDNA sequences are clearly Polynesian (Maori) and show the characteristic, and well documented, Polynesian Motif markers [15], [16]: 16189, 16217, 16247, and 16261 (see Table 1).

**Table 2:** Estimated haplotype (h) and nucleotide (π) diversity.

Population	*N*_ind_	*N*_haplo_	h	Π
European	67	64	0.999	0.00145
Chinese	52	52	1.000	0.00186
Melanesian	50	48	0.998	0.00172
Maori	20	11	0.916	0.00018
All populations	189	174	0.999	0.00174

***N*** **_ind_** (number of sequences), ***N*** **_haplo_** (number of haplotypes), **h** (haplotype diversity), **π** (nucleotide diversity).

### Phylogenetic Analysis

**Figure 2:**
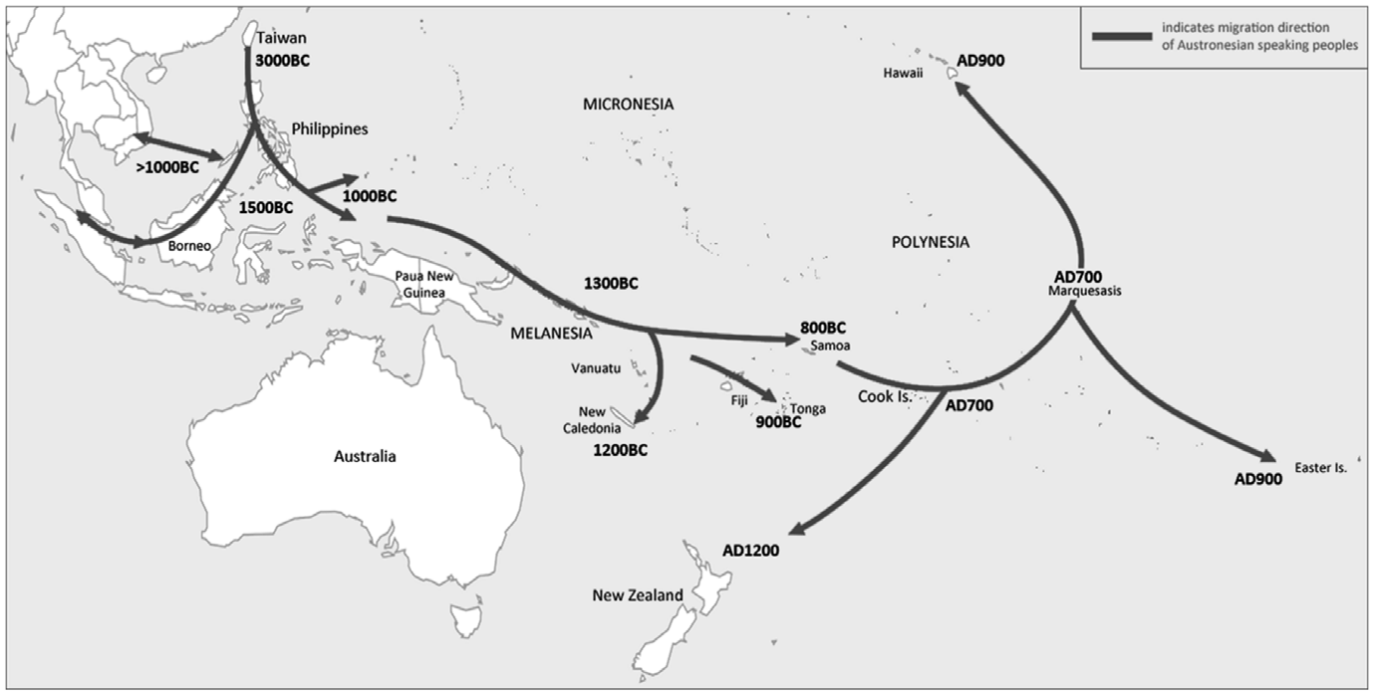
Phylogenetic reconstruction detailing haplogroup B4a1 in complete mtDNA sequences. This neighbour-joining tree was created in MEGA4, using the Tamura-Nei substitution model. The sequences used were sourced from Trejaut et al. (2005), and include the 20 complete Maori mtDNA's. Shown in red are the respective tribes of the Taiwanese Aboriginal sequences.

Phylogenetic analysis of the Maori sequences in the software mtPhy [17] confirmed that all 20 belong to haplogroup B. As expected from previous studies of the Hyper Variable Region (HVR) in Polynesians [11], [12], [15], [18], [19], these Maori sequences all group deep within haplogroup B (for reference see Phylotree [20]). To further investigate the sub-structure of haplogroup B a detailed phylogeny was reconstructed to include 64 complete sequences representing B4a (10 Asian, 14 Taiwanese, 4 Coastal PNG, 16 Pacific Islanders and 20 Maori). Figure 2 illustrates this tree (Asian/Austronesian mt DNA sequences) and shows that all 20 Maori sequences group within B4a1a1, with Pacific Islander and Coastal PNG (Melanesian) mt sequences. These groupings fit well with previous mtDNA work [18], [19], [21], [22], [23], [24], and complement the hypothetical model of Polynesian origin stemming from Taiwan [25]. Apart from the variants which define haplogroup B, we have identified three novel Polynesian (Maori) haplotypes – until now all documented Polynesian mt haplotypes have been B4a1a1a. Table 3 displays the frequency and specific markers for the haplotypes identified in the 20 complete Maori mt sequences. The most interesting haplotype, B4a1a1a3 (unpublished data), was recently included in an updated build of Phylotree (http://www.phylotree.org/ [20]). This haplotype was present in 35% (n = 7) of the individuals sequenced, and is defined by the variants 1185T and 4769A.

**Table 3:** RHAS Maori mt DNA haplotype markers.

Haplotype	*f*	1185	4769	14022	16126	16189	16217	16247	16261
B4a1a1c##	6	.	G	G	C	C	C	.	T
B4a1a1a*	3	.	G	G	.	C	C	G	T
B4a1a1a3 ##	7	T	.	G	.	C	C	G	T
B4a1a1a5##	4	.	G	G	C	C	C	G	T

* Previously reported Polynesian mt DNA haplotype.

## Novel Polynesian (Maori) haplotypes, numbered following Phylotree nomenclature. B4a1a1a3 was included in a recent update to Phylotree. Variants to the CRS are indicated.

### Novel mt DNA sequence variants in Maori

This study has identified six novel (undocumented) mtDNA variants in the Maori sample: five within protein coding regions and one in the control region (see Table 4). An extended database search of mtDB [26] and Mitomap [27] and for these variants returned no hits, thus these positions are deemed to be novel. Two variants result in amino acid sequence changes; 9255T (Pro→Ser) and 15014C (Phe→Leu). Apart from variant 3909T, the novel variants were only noted in individuals and are not present in the wider population and are thus probably sporadic, rather than ancestral.

**Table 4:** Novel mtDNA variants observed in 20 Maori individuals.

Gene	Nucleotide Change	Protein Change	No. Individuals	Percentage
16SrRNA	m.1806T>C	NA	1 (20)	5
ND1	m.3909C>T	Syn	3 (20)	15
COXI	m.6782T>C	Syn	1 (20)	5
COXIII	m.9255C>T	p.MT-COXIII:Pro17Ser	1 (20)	5
Cyt b	m.15014T>C	p.MT-Cyt b:Phe90Leu	1 (20)	5
HVRI*	m.16295C insA	NA	1 (20)	5

* HVRI is non-coding. NA, not applicable; 16SrRNA, 16S ribosomal RNA; ND1, NADH dehydrogenase subunit 1; COXI, cytochrome c oxidase I; COXIII, cytochrome c oxidase III; Cyt b, cytochrome b; HVRI, hyper variable region I.

### Mitochondrial gene variant frequencies in different subpopulations

Estimated frequencies of variants within mitochondrial genes were calculated for the NZ Maori as well as for European, Chinese and Melanesian subgroups, selected because they are each putative ancestral contributor populations of NZ Maori. Of the 13 mtDNA genes, the Maori mtDNA sequences contained variable sites in 9 genes, the majority being population specific polymorphisms (haplogroup B defining variants). There were 19 variants spread across these 9 genes, with *COI*, *ATP6*, and *Cyt b* showing the largest number of variants among the ethnic subgroups. The variant frequency differences between these four groups are displayed in Table 5. Of particular interest in terms of metabolic disease risk was the presence of non-synonymous variants in *COI*, *ATP6*, *COIII* and *Cyt b* genes compared to the ancestral subgroups. The most notable population specific polymorphism, variant A15746G in *Cyt b*, was observed in all 20 Maori samples but was absent or rare in all ancestral subgroups (Table 5). The rare variant, 4769A in *ND2*, is also of particular interest for several reasons; 1) it is a rare polymorphism identified in the CRS [13], yet it is identified at 35% (n = 7) in the NZ Maori cohort, and 2) alongside variant 1185T this variant forms a unique Maori haplotype.

**Table 5:** mtDNA coding variant frequencies in four human populations.

		Rare allele frequency*			
Gene	Variant	European	Chinese	Melanesian	Maori
		(n = 101)	(n = 52)	(n = 56)	(n = 20)
ND1	C3909T	0	0	0	0.15
ND2	G4769A	0.01	0	0	0.35
	T5465C	0	0	0.16	1
COI	G6261A	0.01	0	0	0.10
	T6719C	0	0	0.16	1
	T6782C	0	0	0	0.05
	A6905G	0	0	0.04	0.05
	C7028T	0.63	1	0.98	1
ATP6	G8865A	0	0	0.02	0.10
	G9123A	0	0	0.016	1
	G9145A	0	0	0	0.05
COIII	C9255T	0	0	0	0.05
	T9722C	0	0	0	0.05
ND3	T10238C	0.03	0	0.16	1
ND4	G11719A	0.56	1	1	1
ND5	A14022G	0	0	0.14	1
Cyt b	C14766T	0.57	1	1	1
	T15014C	0	0	0	0.05
	A15746G	0	0	0.16	1

* compared to CRS.

## Discussion

This study provides the first complete mitochondrial sequence data for a Polynesian (Maori) population, and as such allows a rare opportunity to enhance the maternal phylogeny in Oceania as well as explore the mitochondrial genome for potential metabolic risk genes in Polynesians. Although sequence alignment of the Maori mt genomes illustrated high concordance with other Polynesian mt sequences, phylogenetic analysis was able to refine the haplotype substructure of Polynesians. Specifically, Maori mt sequences were deemed as belonging to major mt haplogroup B and formed sub structures within the B4a1a1 ‘haplotype’. This analysis also confirmed the presence of the 9-bp deletion and characteristic control region variants which have become collectively know as the “Polynesian motif” [15]. Identification of these Polynesian informative sites is consistent with previous mt DNA studies in NZ Maori [11], [12].

It has been previously documented that Polynesian and central/eastern Micronesian populations show reduced mtDNA diversity, sharing high frequencies of the single mtDNA haplotype - B4a1a1 [23], [28]. We explored the possibility of decreased mt sequence diversity within the Maori population. Both haplotype (h) and nucleotide diversity (π) were shown to be lower in Maori mt genomes compared to putative ancestral populations. Nucleotide diversity exhibited a 10-fold decrease when compared to three ancestral populations. Evidence of such dramatically reduced diversity of the mt genome in Maori is probably due to founder effects during island hoping migrations and is supported by previous studies [11], [12]. It is perhaps not surprising that due to this reduced mt genetic diversity no unique mtDNA haplotypes have so far been discovered within the Maori population. However, our complete mitochondrial genome scan revealed the presence of at least 3 specific sub-haplotypes of haplogroup B in Maori, which are derived from variants 1185T, 4769A, and 16126C. These three variants could have arisen in the seafaring Polynesian ancestors of Maori, or they could have occurred more recently, i.e. since the settlement of NZ. These variants form a unique mt signature within this Maori population, one that is worth exploring further in other NZ Maori populations to determine its generalizability. As there is very little coding region information available for other Polynesian mt sequences, with only 7 complete Polynesian sequences listed in mtDB [26], it is not currently possible to determine whether these ‘signatures’ are unique to Maori. They may in fact be present, but as yet undetected, in the broader Polynesian population. Nevertheless, these new findings provide a more specific mt ancestry informative marker for future genetic studies involving Maori subjects.

Our results also indicate that the protein coding regions within the mitochondrial genome for the populations of Island Southeast Asia, Coastal Melanesia (PNG), Polynesia, and NZ Maori, which are all mt haplogroup B, are heavily conserved and have not changed much over the ∼5000 years since the suggested movement from Taiwan. The presence of population specific polymorphisms consistent with those previously identified in haplogroup B was confirmed via comparison across four putative ancestral populations. There was one coding variant (4769A) that is not a haplogroup B defining marker which showed increased frequency in the NZ Maori group, although further work is require to accurately confirm it's prevalence in the wider Polynesian community. The lack of coding variation is most likely attributed to genetic drift attributed to the rapid expansion and migration of Austronesian peoples from Taiwan throughout Oceania in the last ∼5000 years [25], [29]. Regardless, our findings make these variants good candidates for future genetic association studies of metabolic disease in Maori populations.

Disease association with specific mtDNA variants has been previously noted for several metabolic traits, including; type-2 diabetes (T2D) [30], [31], [32], [33], [34], insulin resistance [35], [36], [37], and BMI/fat mass [10], [33], [38]. One specific mtDNA variant, 16189C – a fundamental haplogroup B variant, has previously been identified to associate with T2D, insulin resistance and BMI in separate studies [30], [33], [34], [36], [38]. Whether the variant itself is the cause of the association, or simply a marker for the larger haplotype/signature or in linkage with other causal variants located in the nuclear genome is yet to be seen.

In conclusion, this study reports the first complete mitochondrial genome sequence data for a Maori population. Overall, these new data reveal unique mitochondrial genome characteristics in this Polynesian population and enhance the phylogenetic picture of maternal ancestry in Oceania. The presence of several newly identified novel variants, as well as the presence of previously identified disease associated variants, offers plausible candidates for future studies aimed at assessment of metabolic disease risk in Polynesian populations.

## Materials and Methods

### Samples

This project is part of the Rakaipaaka Health and Ancestry Study (RHAS) which is aimed at identifying the genetic and environmental determinants of health in the indigenous Maori tribe (iwi) – Ngati Rakaipaaka. Being a DNA-based genetic study involving indigenous Maori participants the RHAS has taken several years to develop in terms of ethical and cultural approval. The RHAS is governed by Te Iwi o Rakaipaaka (TIORI) in Nuhaka and has received full ethical approval from the Multi-regional ethics committee of New Zealand (MEC/05/12/174). All individuals involved signed a consent form acknowledging they understood the genetic nature of this health research and wished to participate. For this mitochondrial project we selected a subsample of 20 adult individuals who were deemed to be non-admixed (ie. have full Maori ancestry). This was determined by the individual self-reporting that they had four Maori grandparents. Genomic DNA was isolated with the use of commercial kits (FlexiGene – QIAGEN). Polynesian mt DNA ancestry was validated using the previously documented “Polynesian motif”[15] (9-bp deletion plus three control region SNPs), which was found to be present in all 20 DNA(unpublished data).

### mtDNA sequencing of 20 Maori individuals

Complete mitochondrial DNA sequence information was obtained for the 20 Maori individuals using the Mitochip Resequencing Array [39]. The Mitochip Resequencing protocol (Affymetrix, Santa Clara, CA) laid out by Affymetrix was followed (Affymetrix GeneChip CustomSeq Resequencing Array Protocol version 2.1.), and the chips were run on Affymetrix GeneChip equipment (GeneChip Hybridization Oven, GeneChip Fluidics Station, and GeneChip Scanner 3000). The raw data files were analysed using the GeneChip Sequence Analysis Software 4.1 (GSEQ 4.1). Complete mtDNA sequences were exported from GSEQ4.1 and aligned against the revised Cambridge Reference Sequence [13] (CRS) in MEGA4.1 [40]. All sequence data has been submitted to GenBank (awaiting Accession numbers).

### Sequence analysis and mtDNA diversity

Aligned sequences were exported as FASTA files from MEGA4.1, these were then entered into the program mtPhyl [17], where sequence haplotypes and sequence variation statistics were calculated. The mtPhyl software also reported information regarding changes in amino acids and respective position and conservation of these changes. Mitochondrial DNA diversity calculations were performed in DNAspV5 [14] on groups of sequences from four different ethnic populations; European (n = 101) [8], [41], [42], [43], [44], Chinese (n = 52) [42], [43], [45], [46], Melanesian (n = 56) [24], [42], [47], [48], and NZ Maori (n = 20). European, Chinese and Melanesian sequences were obtained from the databases mtDB [26] and PhyloTree [20]. Haplotype (h) and nucleotide (π) diversity statistics were calculated in each ethnic group, as well as in the total sample (all sequences pooled together).

### Phylogeny reconstruction

A consensus neighbour joining tree showing the detail of sub-branching patterns within haplogroup B was reconstructed for a total of 64 complete mitochondrial sequences; all 20 Maori sequences and 44 mitochondrial sequences (Austronesian, Coastal Melanesian and Oceania) obtained from previous studies [25], [32], [47], [49], [50]. The phylogeny was constructed in MEGA4.1 [51] using the Tamura-Nei method and a bootstrap of 500 replicates.

### Novel variants and ‘global’ variant frequencies

Observed mtDNA variants in the Maori sequences were searched against known electronic databases (mtDB [26] and mitomap [27]) to identify potential unreported (novel) DNA sequence variants. Identification of possible thrifty genes in Maori involved comparing mitochondrial gene variant frequencies between candidate ancestral populations: European, Chinese (Asian), Melanesian. Sequences were aligned in MEGA4.1 and variant frequencies between population groups were calculated.
