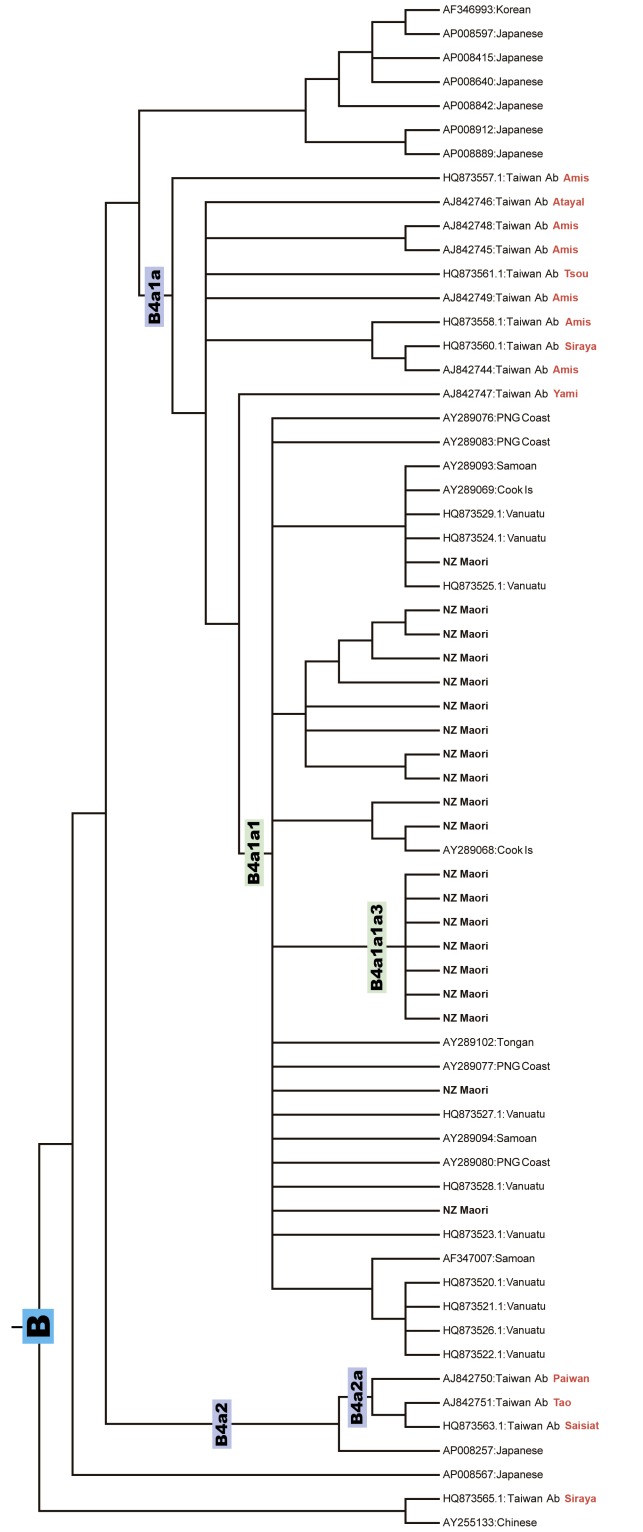# Correction: Complete Mitochondrial Genome Sequencing Reveals Novel Haplotypes in a Polynesian Population

**DOI:** 10.1371/annotation/08aca5be-f760-4595-8562-faf009899c46

**Published:** 2012-05-02

**Authors:** Miles Benton, Donia Macartney-Coxson, David Eccles, Lyn Griffiths, Geoff Chambers, Rod Lea

There was an error in Figure 2. The correct Figure 2 can be viewed here: 

**Figure:**